# Transgene × Environment Interactions in Genetically Modified Wheat

**DOI:** 10.1371/journal.pone.0011405

**Published:** 2010-07-12

**Authors:** Simon L. Zeller, Olena Kalinina, Susanne Brunner, Beat Keller, Bernhard Schmid

**Affiliations:** 1 Institute of Evolutionary Ecology and Environmental Studies, University of Zurich, Zurich, Switzerland; 2 Institute of Plant Biology, University of Zurich, Zurich, Switzerland; United States Department of Agriculture, United States of America

## Abstract

**Background:**

The introduction of transgenes into plants may cause unintended phenotypic effects which could have an impact on the plant itself and the environment. Little is published in the scientific literature about the interrelation of environmental factors and possible unintended effects in genetically modified (GM) plants.

**Methods and Findings:**

We studied transgenic bread wheat *Triticum aestivum* lines expressing the wheat *Pm3b* gene against the fungus powdery mildew *Blumeria graminis* f.sp. *tritici*. Four independent offspring pairs, each consisting of a GM line and its corresponding non-GM control line, were grown under different soil nutrient conditions and with and without fungicide treatment in the glasshouse. Furthermore, we performed a field experiment with a similar design to validate our glasshouse results.

The transgene increased the resistance to powdery mildew in all environments. However, GM plants reacted sensitive to fungicide spraying in the glasshouse. Without fungicide treatment, in the glasshouse GM lines had increased vegetative biomass and seed number and a twofold yield compared with control lines. In the field these results were reversed. Fertilization generally increased GM/control differences in the glasshouse but not in the field. Two of four GM lines showed up to 56% yield reduction and a 40-fold increase of infection with ergot disease *Claviceps purpurea* compared with their control lines in the field experiment; one GM line was very similar to its control.

**Conclusions:**

Our results demonstrate that, depending on the insertion event, a particular transgene can have large effects on the entire phenotype of a plant and that these effects can sometimes be reversed when plants are moved from the glasshouse to the field. However, it remains unclear which mechanisms underlie these effects and how they may affect concepts in molecular plant breeding and plant evolutionary ecology.

## Introduction

The widespread use of genetically modified (GM) plants in agriculture, together with the growing number of different crop species and introduced genes, demands sound environmental risk assessment [1], [2], [3], [4]. Following a tiered approach [5], data from such preliminary risk assessment usually form the basis for extended field trials or lead to the rejection of GM plants from further testing at an early stage [6]. Such studies often focus on the risk that a transgene may not show the desired phenotypic effect if the GM plants are moved from the controlled glasshouse environment to the more variable field conditions. However, few studies have reported potentially unintended phenotypic effects of transgenes in GM plants exposed to a range of realistic environmental conditions [7], [8]. From evolutionary and ecological studies on wild plants it is well known that genotype × environment interactions can be large [9], [10], [11], [12], [13], suggesting that similar interactions might occur in GM plants exposed to different environments, including glasshouse versus field environments. Plant breeders know intuitively that plant performance needs to be tested in realistic agricultural environments and regulatory authorities demand such assessments in their guidelines [14]. Recent studies compared metabolic composition and transcriptional changes in GM Maize grown among environments and *in vitro* and outdoors [15], [16]. They found that differences between GM and control plants in metabolic profiles observed under standardized laboratory conditions were lost in the field. However, whether the same was true for ecological traits was not reported in these studies. Furthermore, a careful search in the literature for replicated and randomized studies about the ecological behaviour of GM and control plants in glasshouse versus field environments did not return any published references.

We therefore used the spring wheat variety Bobwhite SH 98 26 *Triticum aestivum* L. — transformed with the wheat *Pm3b* powdery mildew resistance gene [13] — as a model system to study potential transgene × environment interactions in genetically modified plants. We grew four offspring pairs, each consisting of a GM line and its corresponding non-GM control line under different soil nutrient conditions and fungicide treatment in the glasshouse and the field. Although well studied and not showing any abnormalities in the glasshouse, these plants had never been planted outdoors prior to our experiments. We investigated to what extent the single inserted transgene could influence the disease resistance and overall fitness of our study plants and how these effects were modified by moving the plants from the glasshouse to the field. Since the germination rate of our plants was close to 100% (S. Zeller, unpublished data), agronomical performance traits such as seed yield and seed number were used to indirectly assess changes in plant fitness [17]. We asked the following questions: (i) Does the transgene enhance resistance to powdery mildew *B. graminis* f.sp. *tritici* (DC.) Speer and does it have other phenotypic effects such as fitness costs? (ii) Do we find these effects in all transformed lines or is there line-specific variation? (iii) Can intended and unintended effects of the transgene be influenced by environmental factors and are such effects detectable both in the glasshouse and in the field? We consider this study both as an example of how the ecological behaviour of genetically modified plants can be studied with experimental approaches and how such research can lead to insights into phenotypic effects of inserting a single gene artificially into a plant.

## Materials and Methods

### Genetically Modified Wheat

We used four wheat lines carrying the transgene *Pm3b* in different position on the genome and their respective non-transgenic control lines (null-segregants), each derived from different transformation events [18], [19]. *Pm3b* confers race-specific resistance to powdery mildew and was cloned from hexaploid wheat [13]. The lines were generated by biolistic transformation of spring wheat variety Bobwhite SH 98 26 [20]. The plasmids pAHC17+NotI (*PMI*) and pAHC17+3NotI (*Pm3b*) were used as vectors [21], [22]. After *Not*I (for *Pm3b*) or *Not*I/*Hin*dIII (for *PMI*) digestion, only the desired fragments, but no vector sequences, were co-bombarded into wheat. The *Pm3b* gene was cloned under the control of the *Zea mays* L. (maize) ubiquitin promoter [21] and transformants were selected on mannose-containing media using the phosphomannose isomerase (PMI)-coding gene as selectable marker [23]. After regeneration of T0 transformants, four independent T1 families were selected. From each T1 family, an offspring pair was further propagated consisting of a homozygous transgenic plant (GM lines *Pm3b*#1–4) and a null-segregant, i.e. a plant that did neither inherit the *Pm3b* transgene nor the selectable marker (control lines S3b#1–4). Absence/presence of the transgenes was confirmed by Southern hybridization analysis [24] using probes from the PM3B (bp 1231–1956 as referred to the GenBank accession AY325736) and PMI (bp 271–810 as referred to the GenBank accession AAC74685) encoding region. The GM lines contained the *Pmi* gene as well as one complete copy of *Pm3b*, and in the case of Pm3b#4 an additional fragment, which segregated as a single Mendelian locus in the T1 generation. The null-segregants did not show any hybridization signal with the probes from the *Pm3b* as well as the *Pmi* coding genes. For both transgenic as well as null-segregant lines we can not exclude the presence of fragments from the coding genes or promoter/terminator regions which were not covered by the probes used in Southern blotting. The offspring pairs were multiplied to T4 and used for the glasshouse and field experiments. The seeds used in this study were thus obtained from GM and control lines that had passed through four generations of sexual reproduction. Studies with *Drosophila melanogaster* [25] and *Saccharomyces cerevisiae* [26] showed that a gene's position on the chromosome can influence its expression. We therefore assessed the expression level of the *Pm3b* transgene in the four GM lines by semi-quantitative RT-PCR using RNA isolated from leaves of seedlings grown in the glasshouse (Figure S1). As control for equal amount and quality of template cDNA, the expression levels of the *Mlo* gene [27] were determined.

### Glasshouse Experiment

The glasshouse experiment took place in a climate-controlled glasshouse at the Institute of Evolutionary Biology and Environmental Studies, University of Zurich, Switzerland, from August 2007 to February 2008 (day/night temperature: 21/16 C°; additional light: 14 h/10 h day/night period, daily watering by hand). Seedlings of each line were planted individually into 11-cm square pots containing sterilized soil (Ökohum lawn soil, Ökohum AG, Herrenhof, Switzerland). The design consisted of the four GM and the four control wheat lines crossed with three soil nutrient levels (0, 1 or 2 g of “Osmocote exact mini” per L; Scotts, Waardenburg, The Netherlands). One gram of Osmocote per L corresponded to 13.2 g N, 6.6 g P, 9.1 g K and 1.7 g Mg m^−2^. Natural infection of the wheat plants by powdery mildew occurred 1 month after planting. One half of the experiment was subsequently sprayed with a systemic fungicide specific to mildew (2 ml l^−1^ Opus Top; 83.7 g l^−1^ Epoxiconazol and 250 g l^−1^ Fenpropionazol; Maag Agro AG, Dielsdorf, Switzerland). The active ingredient epoxiconazol blocks fungal cell pathways and activates the plants pathogen defences whereas fenpropionazol blocks two enzymes that are related to the fungal cell-wall synthesis. We used a high fungicide concentration (2ml/l); this caused slight leaf chlorosis on several plants that disappeared after a few days. All tested lines were affected equally. Each of the 8×3 line-by-nutrient level combinations was replicated five times. Plants were harvested 162 days after the start of the experiment.

### Field Experiment

The field experiment took place at an agricultural research station in Zurich-Reckenholz, Switzerland. It started in March 2008 and lasted until August 2008. Four replicate blocks, each with sixteen 1×1.08 m plots, were sown with seeds of the same eight wheat lines as used in the glasshouse experiment. In each plot, 400 seeds were sown in six rows with a distance of 18 cm between rows using an Oyjord plot drill system (Wintersteiger AG, Ried, Austria). Fertilizer was applied at the phenological stage 11 and 39 (Zadoks *et al.* 1974) to half of the plots (two times 3 g N m^−2^ as “Ammonsalpeter 27.5”, Lonza, Visp, Switzerland).

The natural field soil provided the plants with sufficient phosphorous, potassium and magnesium (80, 235 and 234 mg kg^−1^). All plots were sprayed with the herbicide cocktail Concert SX (40% Thifensulfurone, 4% Metusulfurone-methyl; Stähler Suisse AG, Zofingen, Switzerland) and Starane super (120 g l^−1^ Bromoxynil, 120 g l^−1^ Ioxynil, 100 g l^−1^ Fluroxypyr-metilheptil-ester; Omya Agro AG, Safenwil, Switzerland) in the beginning of May. In each plot, five individual plants were marked shortly after germination. Powdery mildew and ergot *Claviceps purpurea* (FR.) TUL. infection occurred naturally. Vandals damaged 53 of the 64 plots at random by removing the tops of some plants early in the flowering stage. The damage-induced loss of leaf area was within the natural variation observed in the field and smaller than the herbivory caused by *Oulema melanopus* L. (cereal leaf beetle). The damaged plots recovered within 2–3 weeks and regained their original height and vegetative mass. We recorded the exact area of damage within each plot and replaced all marked plants that had suffered damage (46.3%). A second field experiment with the same plant lines was carried out in an adjacent field the following year. Although plants grew higher because of more favourable weather conditions, the different wheat lines performed very similar as in the 2008 trials (S. Zeller *et al.*, unpublished data). We are therefore confident that the here presented results and conclusions were not influenced by this disturbance.

### Response Variables

We assessed the degree of powdery mildew infection [28] and the phenological stage [29] 80 days after planting. Plants with visible powdery mildew colonies on all their leaves (including flag leaf) were considered infected. We defined plant height as the highest point of the plant measured from the soil and recorded it at the end of the growing season. For these three variables, powdery mildew infection, phenological stage and plant height, we used the maximum values of all tillers per pot or of the five marked plants per plot in glasshouse or field experiment, respectively, for analysis. After ripening, all plants were cut at ground level and separated into vegetative and reproductive parts (spikes). These were then dried at 80 and 25 C°, respectively, and weighed. We then threshed the reproductive parts, counted and removed the seeds infected by ergot (only in field trial) and obtained the total seed mass which is equivalent to the seed yield. The seed number was calculated from the seed yield divided by the average seed mass. The latter was determined on a sample of seeds, one spike in the glasshouse or 1,000 seeds from all spikes in each 1×1.08 m plot in the field. The vegetative mass, seed number and seed yield were total measurements of all plants growing in a pot or a plot. Ergot infection rate was calculated as percentage of seed number.

### Data Analysis

In a factorial design, we grew the eight wheat lines under different fertilizer treatments (three levels in the glasshouse and two in the field). There were five blocks in the glasshouse and four in the field. We analysed the data of both experiments separately and in combination by analysis of variance (ANOVA). The critical significance level was 0.05 in all analyses. All quantitative pot data from the glasshouse were multiplied by 82.64 to equal an area of 1 m^2^. Quantitative field data were divided by 1.08 for the same reason. Regression analysis showed that two variables were slightly affected by the act of vandalism (seed yield: R^2^ = 0.167 and seed number: R^2^ = 0.094; n = 64). We removed this effect by multiplying the data of the damaged plots with the negative slope from the regression analysis multiplied by the degree of damage (for 10% damaged area: seed yield: –1.003 g; seed number: –20.8). We used the statistical software GenStat (VSN International Ldt.) to fit multiple regression models and summarize the results in ANOVA tables for all variables except powdery mildew infection (see Tables S1, S2 and S3). Residual plots were examined to identify outliers and to check if the assumptions of normality and homoscedasticity were fulfilled. The vegetative mass of one unusually heavy plant was identified as an outlier and excluded from the analysis. Phenological stage was transformed to the fourth power (y^4^); vegetative mass, seed yield and seed number were square-root transformed; and ergot infection rate was cube-root transformed. The binary mildew infection data were analysed using multiple logistic regression with analysis of deviance [30].

## Results

### Glasshouse Experiment

**Figure 1:**
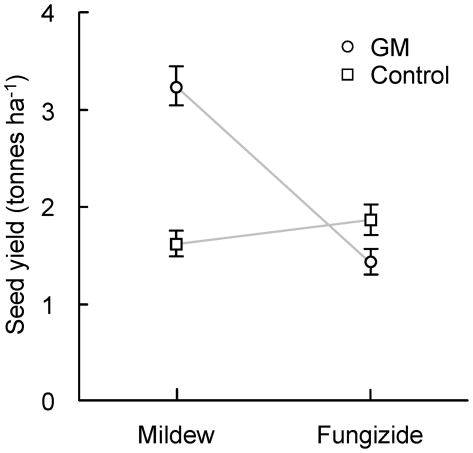
Effects of mildew infection and fungicide spraying on yields of GM wheat lines. Example of significant transgene × environment (presence/absence of powdery mildew) interaction in GM spring wheat in a glasshouse experiment. GM plants (circles  =  *Pm3b*#1 to #4) have higher yield than control plants (squares  =  S3b#1–4) in the presence but lower yield in the absence of mildew (fungicide spraying); light grey lines were drawn to make interactions between transgene and environments visible; error bars represent ±1 standard error (back-transformed from square root scale).

One half of the replicates in the glasshouse experiment were sprayed with fungicide to simulate environments with and without powdery mildew. While the control lines benefited from the fungicide treatment, the GM lines reacted negatively (*P*<0.001 for GM/control x fungicide interaction). The yield of the GM lines dropped lower than the yield of the sprayed control lines (Figure 1). This indicates that the cost of resistance might be high if the pathogen is absent. Furthermore, sprayed plants showed an acute stress reaction in form of chlorotic leaves. We decided therefore to exclude the sprayed portion of the experiment from further analysis.

**Figure 2:**
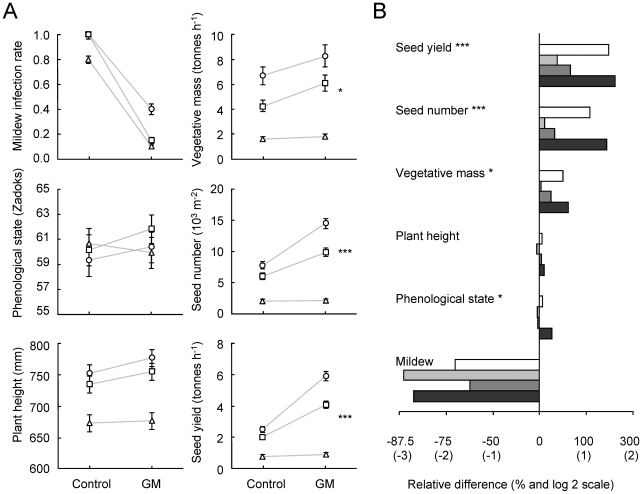
Effects of the transgene in the glasshouse on mildew infection and plant performance traits. The mildew infection equals the proportion of pots with strong powdery mildew infection up to flag leaves. Phenological stage, plant height, vegetative mass, seed number and seed yield were measured to assess the plant performance. A: mean of four lines (Control  =  S3b#1–4; GM  =  Pm3b#1–4) at different soil nutrient levels (circles  =  high fertilizer, squares  =  medium fertilizer, triangles  =  no additional fertilizer); significant transgene × fertilizer environment interactions indicated by asterisks (vegetative mass: *P* = 0.035, seed number: *P*<0.001, seed yield: *P*<0.001); light grey lines were drawn to make these interactions visible; error bars represent ±1 standard error (back-transformed, see methods) and are sometimes hidden behind the symbols. B: proportional difference between GM and control plants for each of the four offspring lines but averaged across nutrient levels (white bars  =  offspring pair 1 (Pm3b#1 *vs.* S3b#1), light grey  =  offspring pair 2, dark gray  =  offspring pair 3, black bars  =  offspring pair 4); x-axis log-scale with original values (100 * GM/control); bars extending to the right from the vertical zero line indicate higher values in GM than in control plants; significant GM/control x offspring pair interactions indicated by asterisks (* *P*<0.05; ***P<0.001).

The *Pm3b* transgene had the desired phenotypic effect and increased resistance to powdery mildew in the glasshouse experiment (Figure 1; *P*<0.001 for difference GM/control plants, see Table S1). The yield of the GM lines doubled (from 1.60 to 3.23 tonnes per ha^−1^) compared to the susceptible control lines. GM plants had also more seeds and higher vegetative biomass than control plants in the glasshouse (Figure 2; both *P*<0.001; see Table S2). Phenological development and plant height were not affected by the transgene, indicating that these traits may be genetically more constrained than the other traits.

The four offspring pairs differed significantly from one another in the five fitness-related traits (phenological stage: *P*<0.001, plant height: *P*<0.001, vegetative mass: *P* = 0.006, seed number: *P* = 0.004, seed yield: *P* = 0.014 for main effect of offspring pair). Alternatively, we tested if there was a significant difference between the four control lines. They differed indeed in all traits except the mildew resistance (phenological stage: *P*<0.001, plant height: *P*<0.001, vegetative mass: *P*<0.001, seed number: *P*<0.001, seed yield: *P*<0.001 for the contrast among offspring lines within control). These differences may be caused by the callus culturing of GM and control lines or effects of the transformation itself. Heritable effects acquired in cell culture can have a genetic basis and plants with such effects are sometimes used in plant breeding [31], [32].

Depending on the offspring pair, the inserted transgene had significantly different effects on three of the measured traits (Figure 2B; vegetative mass: *P* = 0.012, seed number: *P*<0.001, seed yield: *P*<0.001 for GM/control × offspring pair interaction). This suggests that unintended phenotypic effects of the transgene depended on the location where it had been inserted into the genome. In absolute numbers, line *Pm3b*#4 had the highest yield (4.19 tonnes per ha^−1^) of the four tested GM lines and proved to be highly resistant to powdery mildew (only 20% of plants infected).

Fertilizer application in the glasshouse had positive effects on all traits except phenological stage (Figure 2A). Fertilization also increased mildew infection (*P* = 0.016) which might be due to the increased growth rate of the host plant [33]. Increased nutrient content of the plant material could have boosted the spread of mildew directly [34]. Differences between GM and control plants generally increased with nutrient level (vegetative mass: *P* = 0.035, seed number: *P*<0.001, seed yield: *P*<0.001 for fertilizer × GM/control interaction). We currently have no explanation for this result which demonstrates the importance of testing effects of transgenes across a range of environments.

### Field Experiment

**Figure 3:**
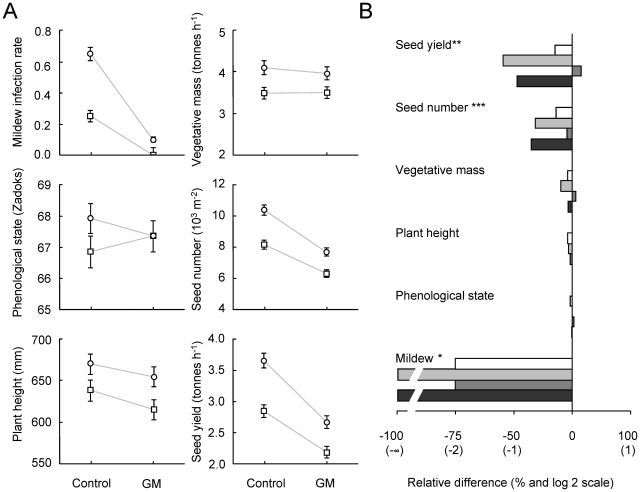
Effects of the transgene in the field on mildew infection and plant performance traits. The mildew infection equals the proportion of pots with strong powdery mildew infection up to flag leaves. Phenological stage, plant height, vegetative mass, seed number and seed yield were measured to assess the plant performance. A: mean of four lines at different soil nutrient levels (circles  =  additional fertilizer, squares  =  no fertilizer); transgene × fertilizer environment interactions were never significant; light grey lines were drawn to make this visible; error bars represent ±1 standard error (back-transformed, see methods). B: proportional difference between GM and control plants for each of the four offspring lines but averaged across nutrient levels (white bars  =  offspring pair 1 (Pm3b#1 *vs.* S3b#1), light grey  =  offspring pair 2, dark gray  =  offspring pair 3, black bars  =  offspring pair 4); x-axis log-scale with original values (100 * GM/control); bars extending to the right from the vertical zero line indicate higher values in GM than in control plants; significant GM/control x offspring pair interactions indicated by asterisks (* *P*<0.05; ** *P*<0.01; ***P<0.001).

**Figure 4:**
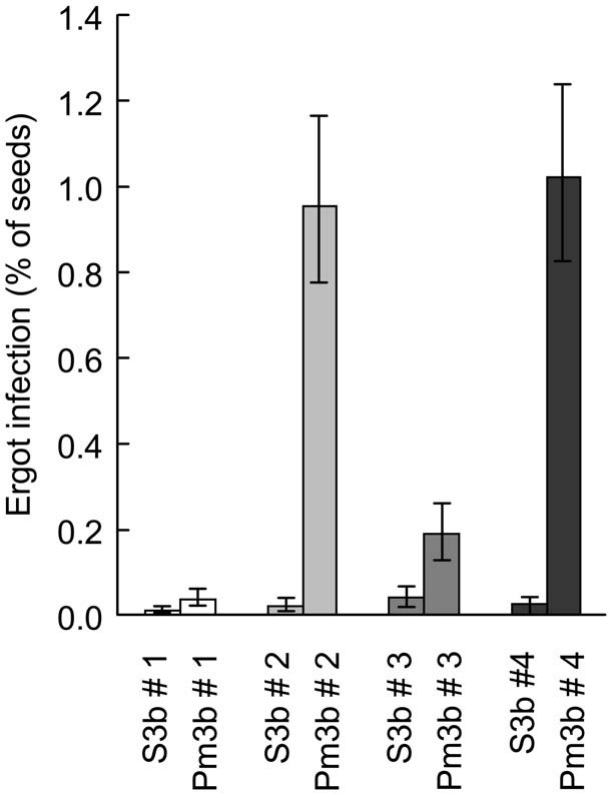
Percentage of ergot infected seeds in GM and control plants in the field. White bars  =  offspring pair 1, light grey  =  offspring pair 2, dark grey  =  offspring pair 3, black bars  =  offspring pair 4. Within each pair, the bar to the left shows control line and the bar to the right shows the corresponding GM line. Error bars represent ±1 standard error (back-transformed from cube root scale.

We measured the same traits in the field experiment as in the glasshouse experiment. In addition we recorded infection by ergot fungus, which occurred naturally in the field but not in the glasshouse. Again, we compared first the four GM lines (Pm3b#1–4) with the control lines (S3b#1–4), then the offspring pairs among each other and finally tested the interaction between these two main effects. GM plants with the *Pm3b* transgene showed increased resistance to powdery mildew (Figure 3A and B; *P*<0.001; see Table S1). In contrast to the glasshouse findings, GM plants had significantly fewer seeds and lower seed yield than control plants (Figure 3A; both *P*<0.001; see Table S3). Phenological stage, plant height and vegetative mass were not affected by the transgene. In the field, GM plants showed increased infection by ergot fungus compared with control plants (Figure 4; P<0.001).

The four offspring pairs differed in seed number and their level of ergot infection (seed number: *P* = 0.004, ergot infection: *P*<0.001 for main effect of offspring pair). Effects of the inserted transgene differed among the four offspring pairs for the dependent variables powdery mildew resistance, ergot infection, seed number and seed yield as reflected in significant GM/control × offspring pair interactions (Figure 3B; powdery mildew infection: *P* = 0.022; ergot infection: *P*<0.001; seed number: *P*<0.001, seed yield: *P*<0.001). That is, in the field, yields of the GM lines *Pm3b*#2 and #4 were reduced by 56% and 48%, respectively, when compared with the corresponding control lines within offspring pairs. The lines *Pm3b*#2 and #4 were completely resistant to powdery mildew in the field, whereas 12.5% of the *Pm3b*#1 and #3 plants were infected. The difference in ergot infection between GM and control lines was small in offspring pair 1 (Figure 4), moderate in offspring pair 3, and large in offspring pairs 2 and 4. Seed infection rates of around 1%, as found in lines 2 and 4, can reduce grain quality.

In the field, fertilization increased plant height (*P* = 0.006), vegetative mass (*P* = 0.003), seed number (*P*<0.001) and seed yield (*P*<0.001). The development of the plants (phenological stage) was not affected by fertilizer application. Similar to the glasshouse, mildew infection increased with fertilizer application in the field (*P*<0.001). However, in contrast to the glasshouse, fertilization did not alter the difference between the GM and control lines in the field.

### Comparison between Glasshouse and Field Experiment

To test if the observed differences in transgene effects between glasshouse and field were statistically significant we also analyzed the datasets from the two experiments together, considering the medium and high nutrient levels in the glasshouse as equivalent to the low and high levels in the field, respectively. As expected, glasshouse and field environments differed significantly from each other. Powdery mildew seemed to favour glasshouse conditions which lead to a stronger infection of the plants in the glasshouse than in the field (*P*<0.001) thus increasing the potential benefits of resistance caused by the transgene in the glasshouse. Glasshouse plants developed more slowly (phenological stage: *P*<0.001) and invested slightly more into vegetative mass (*P* = 0.042) but had fewer seeds (*P*<0.001) and lower seed yields (*P*<0.001) than field plants.

GM plants had a fitness advantage over control plants in the glasshouse, but a disadvantage in the field (vegetative mass, seed number and seed yield: *P*<0.001, plant height: *P*<0.05 for glasshouse/field × GM/control interaction). While the differences between glasshouse and field could not be assigned to a single environmental factor, the different fertilizer treatments (three levels in the glasshouse and two in the field) did represent such a controlled environmental gradient. We found that fertilizer had similar phenotypic effects in glasshouse and field environments.

## Discussion

### Transgene × Environment Interactions

This study demonstrates that GM plants can differ in morphological, fitness- and pathogen-related traits from their control plants. We found several significant transgene (GM vs. control) × environment interactions; that is, depending on the environmental conditions the studied transgene against mildew infection had beneficial or detrimental effects on most of the investigated plant traits. GM plants generally benefited from glasshouse conditions with high mildew infection pressure when compared with control plants but showed a stress reaction when powdery mildew was absent due to fungicide spraying. It is possible that the GM plants lacked the energy to cope with the stress caused by this treatment or the chemical itself could have interacted with the transgene or with pathways involved in *Pm3b*-mediated resistance. It is conceivable that the high fungicide dose increased the extent of the stress reaction of GM plants.

Similar to the fungicide treatment in the glasshouse, the natural conditions outdoors seemed to have stressed the GM plants in the field to the extent that their fitness was significantly reduced. Possible causes of environmental stress in the field were drought and neighbour competition. The only deliberately manipulated factor, i.e. fertilizer application, modified the transgene effects only in the glasshouse but not in the field. Apparently the transgene only offered a relative fitness benefit to GM plants growing under conditions of high mildew incidence but low levels of other stresses. These were exactly the conditions met in the glasshouse but not in the field (nor in the glasshouse after fungicide application). Under less beneficial conditions, the GM plants may have paid a physiological cost for the high intrinsic mildew resistance [35].

### Differences among GM Lines

The four GM lines, which each contained a single copy of the identical transgene in homozygous condition, differed significantly from each other. There are several potential reasons for these differences. It is possible that cell culturing caused somaclonal variation among the four offspring pairs which subsequently might have interacted differentially with the transgene [32]. Although theoretically possible [36] we would not expect that such interactions would be stably inherited over five plant generations as we found it here. It seems unlikely that random somaclonal events would cause similar effects in two of the four independently transformed lines (*Pm3b*#2 and #4). A more plausible explanation for the differential effects of the inserted transgene among the four offspring pairs may be that positional effects caused the line-specific differences. Several processes are known to cause such effects [37]. Firstly, an inserted transgene may disrupt native genes. Because spring wheat is hexaploid, consists of more than 80% repetitive, non-genic DNA sequences and each GM line was created by a single insertion event, it is unlikely that the disruption of coding genes or their regulatory sequences could have caused these differential effects [38], [39]. Secondly, the insertion position of a transgene into the genome may have affected its expression level. Studies have shown that transgene expression rates and activity patterns of independently transformed wheat lines with constitutive ubiquitin promoters can vary [40]. Depending on the insertion site, flanking DNA regions may partially silence the inserted promoter. Head-to-tail arrangements of the transgenes, in our case of the *Pm3b* and the selectable marker gene, could also have a negative influence on the promoter activity [41]. It is also possible that in some lines the transgene was inserted into a region of the genome with low transcription activity [42].

The semi-quantitative expression analysis (Figure S1) indicated that the expression of the *Pm3b* transgene did differ between the four GM lines. Thus, although we lack confirmation by quantitative expression data, it appears that the two GM lines *Pm3b*#2 and #4, where the transgene showed the strongest phenotypic effects, also had the strongest transgene expression. Obviously, this hypothesis should be tested with a much larger number of lines differing in expression levels. However, such a study currently would be beyond our capacities to obtain funding and permissions for field trials. If the hypothesis could be confirmed, there would still be the question whether the overexpression of the transgene led to an overabundance of its protein product and the subsequent phenotypic effects or if other mechanisms would be involved.

Besides the quantitative reduction of fitness, we observed that some spikes of the two lines *Pm3b*#2 and #4 also differed in their morphology during flowering time and that the same two lines were also more heavily infected by ergot fungus than the other two GM lines and the four control lines. The altered spike morphology may have increased the likelihood of ergot spores entering the florets [43]. However, no indications of altered spike morphology were observed in the glasshouse.

### Implications for Molecular Plant Breeding

Although transgenic plant lines with unintended phenotypes commonly arise during molecular plant breeding [4], [37] they can usually be detected earlier and more easily and are thus not further investigated [3] and published. The development of commercial GM plants is based on long selection processes that start in the glasshouse and end in the field. Enormous numbers of seedlings are already discarded before they are exposed to realistic field settings. Our results may have implications for molecular plant breeding: some of the best GM lines in the glasshouse may still show aberrant performance in the field and some not so promising GM lines in the glasshouse may actually be the best for the field. They would likely be lost at early stages of a selection process only targeted at maximum performance under a particular environment. Based on our glasshouse findings, line *Pm3b*#1 would have suffered this fate yet was the best in the field. One lesson from our study and from genotype × environment studies in general [9], [10], [11], [44] is that lines which perform particularly well in a specific environment may pay a cost of specialization and perform poorly in other environments.

### Conclusions

Our study demonstrates that inserting a single transgene into the hexaploid wheat genome, along with the desired target effect such as mildew resistance in the present case, can significantly affect other phenotypic traits and thus, as in our case, change the ecological behaviour of the species (hypothesis (i) in Introduction). Such unintended effects of single genes to our knowledge are always smaller in experiments using naturally occurring genetic variation and wild plants [45], [46]. Even when we included crop plants, we could not find any publications where single genes reduced quantitative fitness traits in a plant as strongly as in the present case, yet only in the field and not in the glasshouse [47]. Commercial glyphosate-resistant soybean cultivars were found to suffer from a 5% yield depression that might be caused by the transgene or its insertion process [48]. One study tested wheat varieties with introduced resistance genes against leaf and stripe rust and reported a 12% reduction of yield [49], which was considered to be a very large effect [50]. Compared with these, the yield reductions of 48 and 56% observed in our two GM lines of wheat expressing the *Pm3b* transgene are much larger (Figure 3B).

We found that the level of mildew resistance as well as the magnitude of other phenotypic effects varied significantly between different GM lines (hypothesis (ii) in Introduction). We hypothesize that this variation in phenotypic effects may be due to different expression levels of the *Pm3b* transgene which in turn might have been caused by different insertion positions of the transgene in the genome. Some plant breeders suggest not selecting for plant lines with complete pathogen resistance because costs of such a resistance often outweigh benefits [47]. In our case this would speak for selecting GM lines with relatively low expression levels yet still increased mildew resistance, i.e. line *Pm3b*#1 [51]. However, to test the hypothetical correlation between expression level and phenotypic effects would require specific experiments with a larger number of GM lines as used here. With regard to risk assessment our findings are in agreement with the view that a each GM line should be tested in a case-by-case approach [52].

Finally, our results show that even if desired phenotypic effects of a transgene are found across a range of environments in a glasshouse experiment, some of these effects can be reversed if GM lines are exposed to natural environmental variation in the field (hypothesis (iii) in Introduction). Although it is likely that commercial plant breeders know of the presence of transgene × environment interactions, it seems that such observations so far have not found their way into the scientific literature. Breeding trials to select lines for further investigation do not need full replication and randomization, yet for an assessment of the ecological behaviour of such lines, replicated and randomized ecological experiments would be required. Our study may serve as an example of potential results that can be obtained in such experiments. We believe that such experiments can help us to gain a deeper understanding of single-gene effects in plant ecology and evolution.

## Supporting Information

Figure S1 Semiquantitative expression analysis of *Pm3b* and *Mlo* in GM wheat lines.
(0.44 MB DOC)

Table S1 ANOVA tables of powdery mildew infection data from glasshouse and field experiments.
(0.05 MB DOC)

Table S2 ANOVA table of phenological state, plant height, vegetative mass, seed number and seed yield data from the glasshouse experiment.
(0.05 MB DOC)

Table S3 ANOVA table of phenological state, plant height, vegetative mass, seed number, seed yield and ergot infection data from the field experiment.
(0.04 MB DOC)
