# Supplementary material for: Transgene × Environment Interactions in Genetically Modified Wheat
Source: PLoS One. 2010 Jul 12;5(7):e11405. doi: 10.1371/journal.pone.0011405 (PMC2902502; doi:10.1371/journal.pone.0011405)
Supplement: Figure S1 — Semiquantitative expression analysis of Pm3b and Mlo in GM wheat lines. (0.44 MB DOC) [file pone.0011405.s001.doc]

**Figure S1.** **Semiquantitative expression analysis of *Pm3b* and *Mlo* in GM wheat lines.**

30 cycles

28 cycles

25 cycles

35 cycles

30 cycles

25 cycles


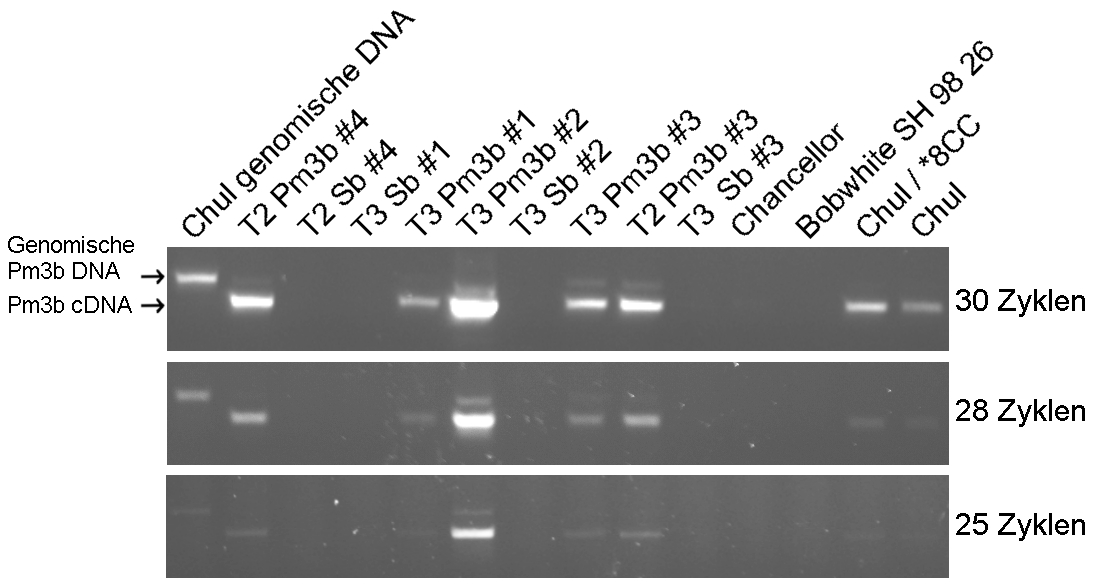

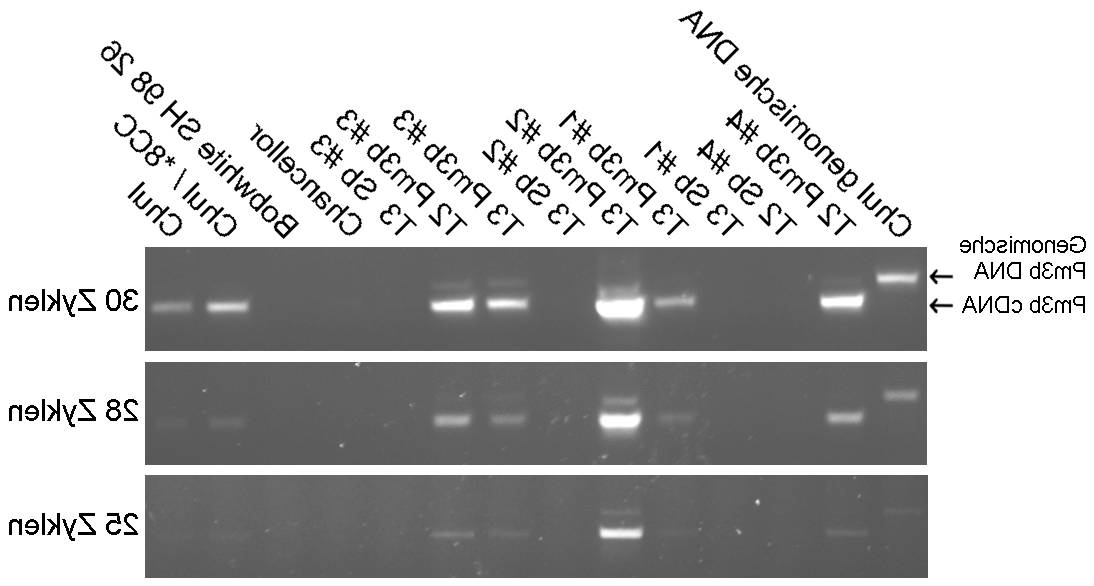

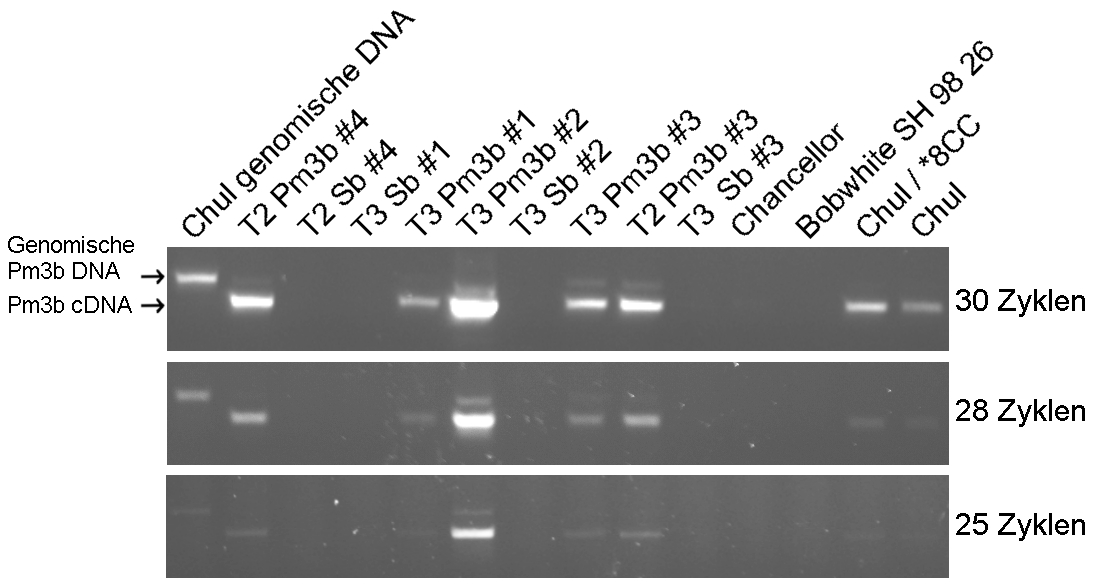

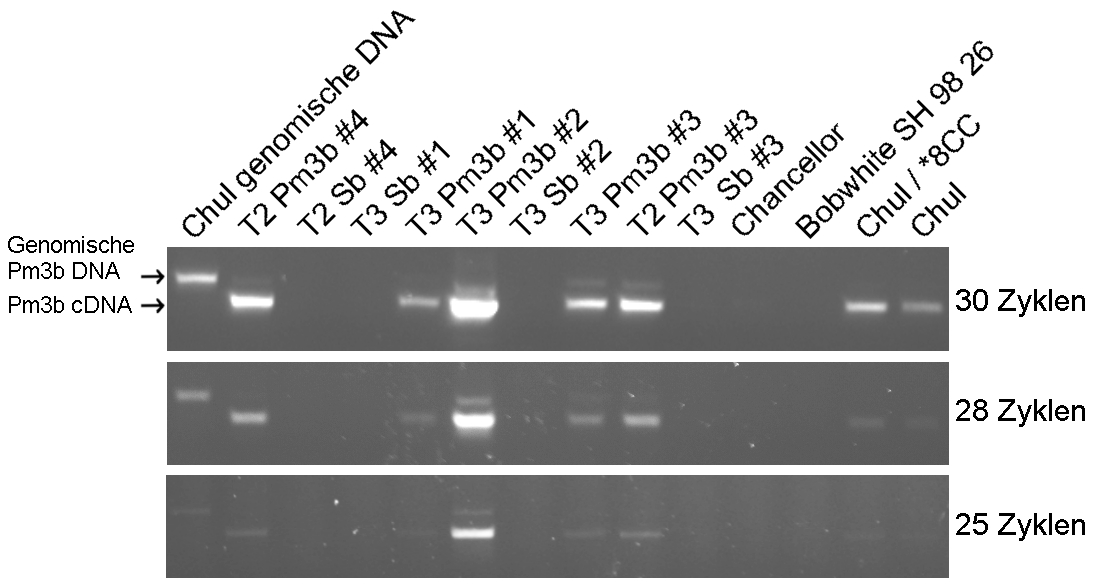

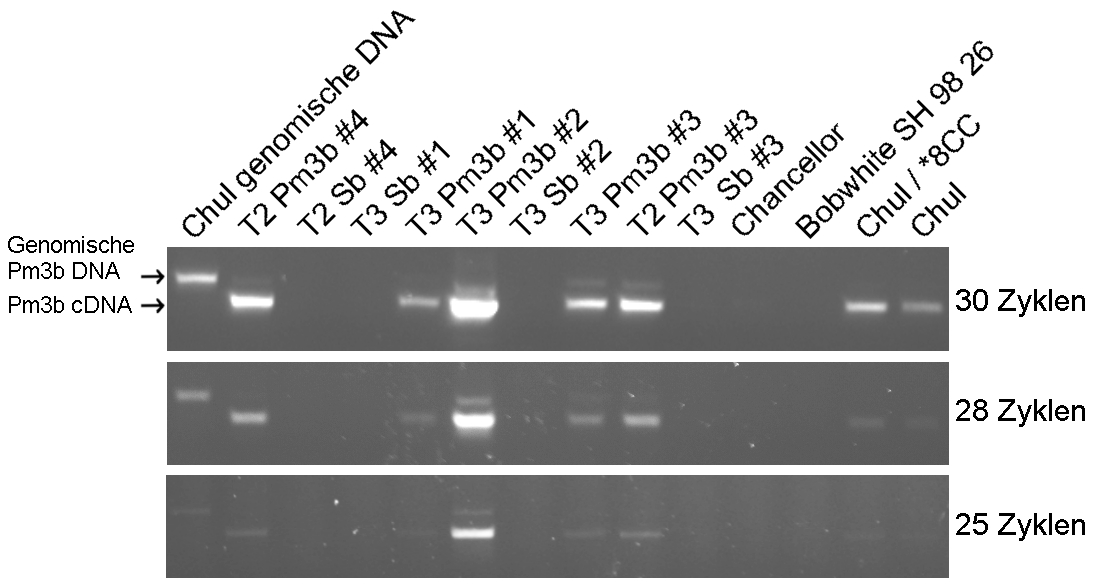

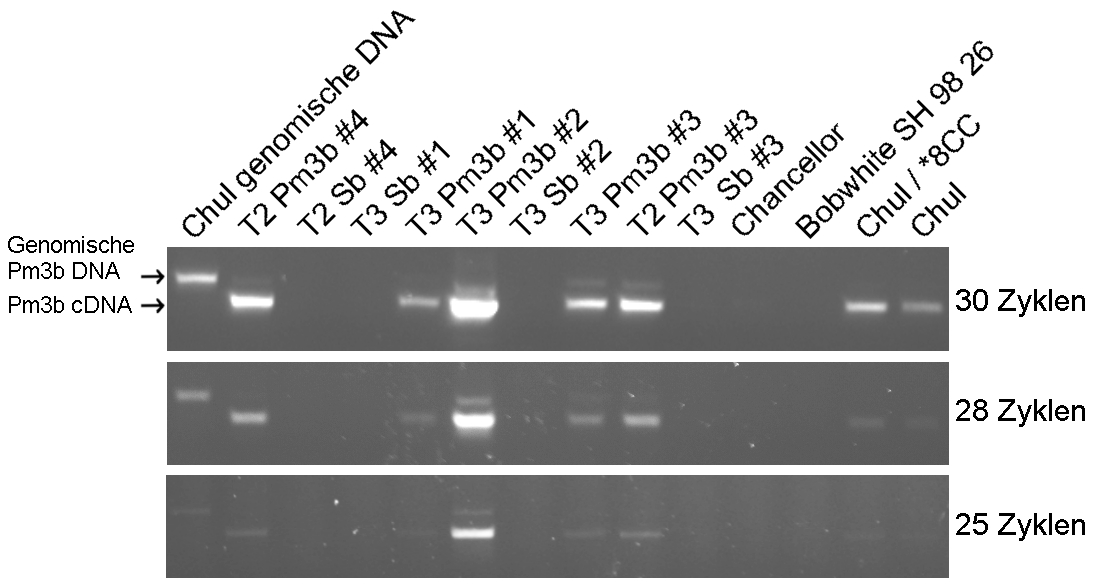

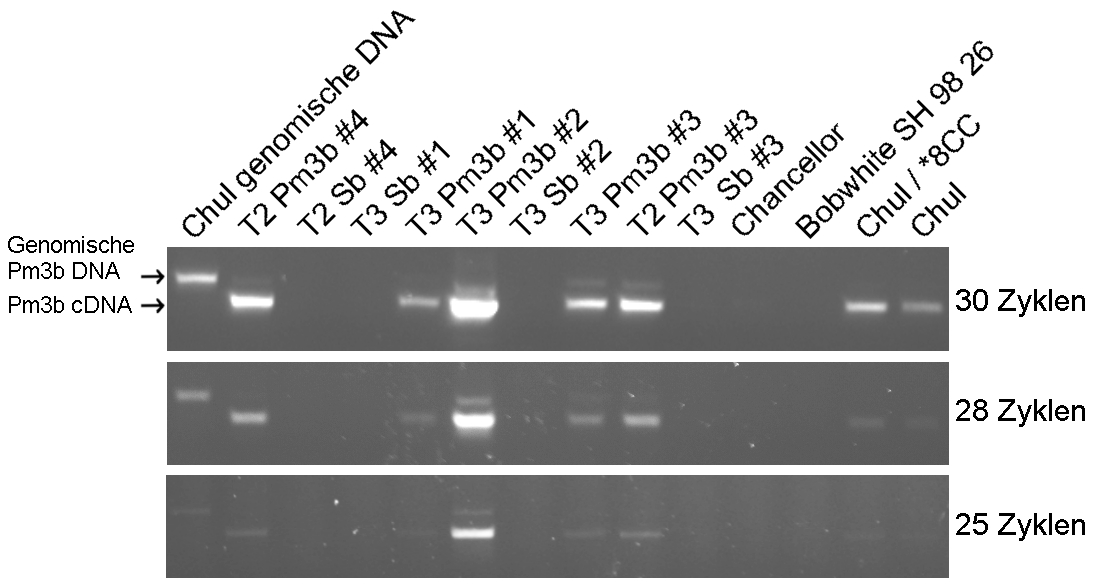


a

b

c

d

e

f

g

h

i

j


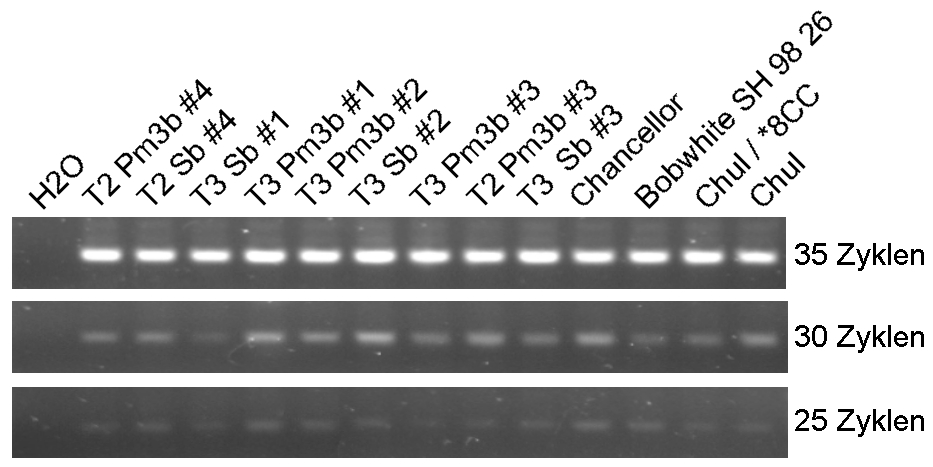

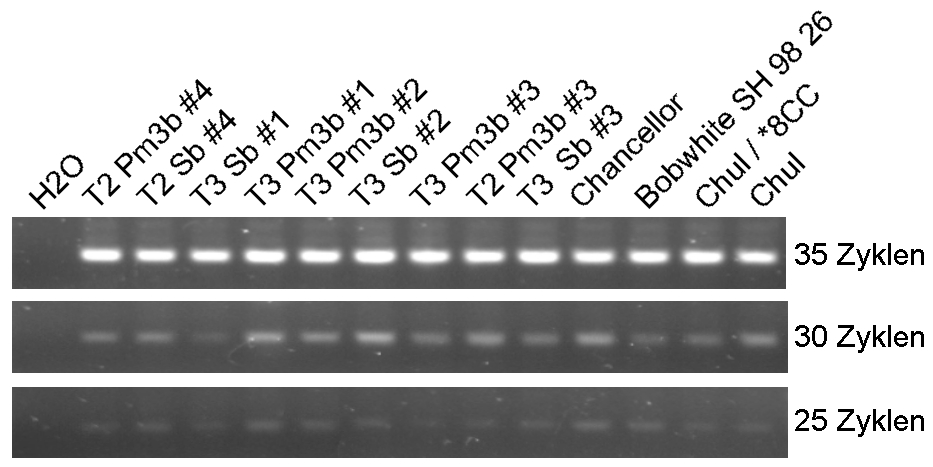

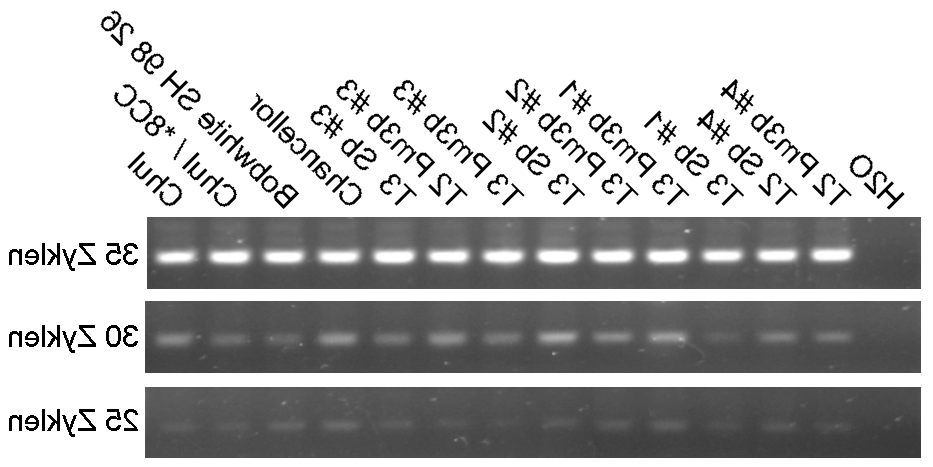

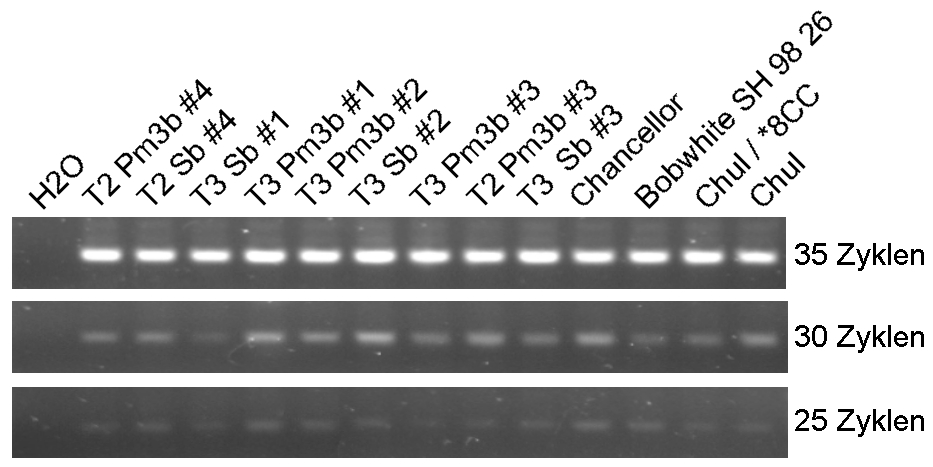

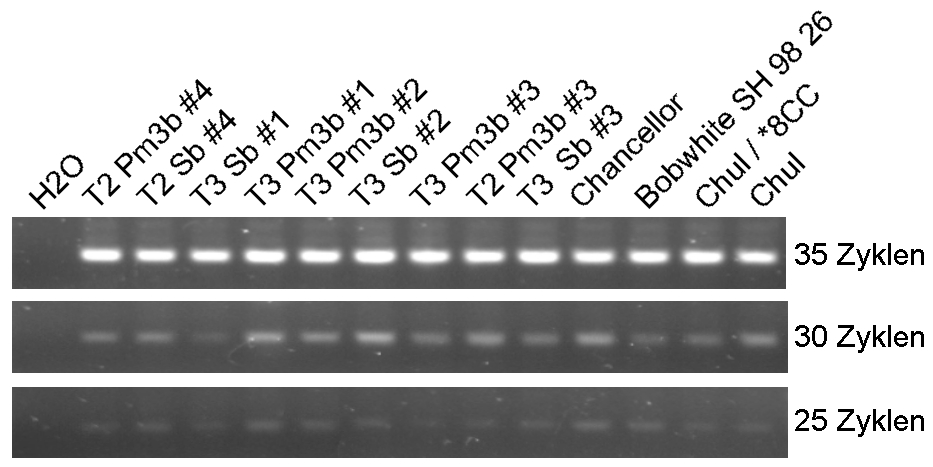

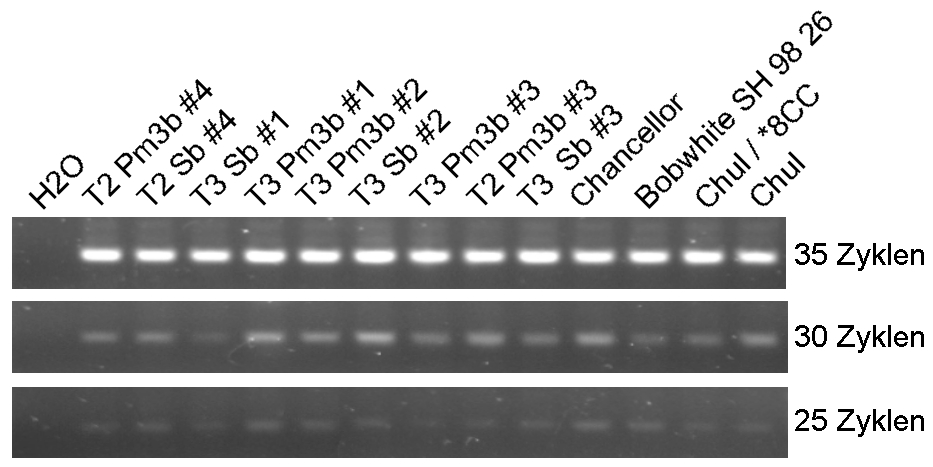


a

b

c

d

e

f

g

h

i

j

A

B

These gel photographs show semi-quantitative PCR expression analyses. A: Analysis of *Pm3b* expression in the *T. aestivum* lines *Pm3b* #1-4 (b, d, f, h) and the corresponding control lines S3b #1-4 (c, e, g, i). As positive controls, genomic DNA (a) and cDNA (j) of the variety Chul carrying one endogenous copy of *Pm3b* were used. The number of PCR cycles is indicated on the right. The photographs of the gel were cropped and rearranged graphically. B: As control for equal amount and quality of template cDNA, the expression levels of the *Mlo* gene were determined. Negative control water (a), *Pm3b* #1-4 (b, d, f, h), corresponding control lines S3b #1-4 (c, e, g, i), variety Chul (j).
