# Supplementary material for: Transgene × Environment Interactions in Genetically Modified Wheat
Source: PLoS One. 2010 Jul 12;5(7):e11405. doi: 10.1371/journal.pone.0011405 (PMC2902502; doi:10.1371/journal.pone.0011405)
Supplement: Table S1 — ANOVA tables of powdery mildew infection data from glasshouse and field experiments. (0.05 MB DOC) [file pone.0011405.s002.doc]

**Table S1.** This ANOVA table shows the effect of the Fertilizer, GM / control, Offspring pair treatments and their interactions on the phenological state, plant height, vegetative mass, seed number and seed yield in the glasshouse experiment.

|  |  | Phenological state | | Plant height | | Vegetative mass | | Seed number | | Seed yield | |
| --- | --- | --- | --- | --- | --- | --- | --- | --- | --- | --- | --- |
| Source of variation | df | % SS | F pr. | % SS | F pr. | % SS | F pr. | % SS | F pr. | % SS | F pr. |
| Block | 4 | 7.1 | 0.018 | 0.4 | 0.933 | 0.2 | 0.534 | 0.7 | 0.374 | 0.6 | 0.379 |
| Fertilizer | 2 | 0.5 | 0.651 | 26.3 | <.001 | 86.1 | <.001 | 61.8 | <.001 | 55.6 | <.001 |
| GM / control | 1 | 0.3 | 0.500 | 1.2 | 0.142 | 2.8 | <.001 | 8.2 | <.001 | 16.3 | <.001 |
| Offspring pair | 3 | 26.4 | <.001 | 15.8 | <.001 | 1.0 | 0.006 | 2.5 | 0.004 | 1.6 | 0.014 |
| GM / control x Offspring pair | 3 | 6.0 | 0.017 | 3.4 | 0.099 | 0.9 | 0.012 | 4.7 | <.001 | 3.9 | <.001 |
| Fertilizer x GM / control | 2 | 0.6 | 0.586 | 0.4 | 0.720 | 0.5 | 0.035 | 4.1 | <.001 | 6.8 | <.001 |
| Fertilizer x Offspring pair | 6 | 3.5 | 0.410 | 0.6 | 0.979 | 0.5 | 0.394 | 0.9 | 0.536 | 0.5 | 0.717 |
| Fertilizer x GM / control x Offspring pair | 6 | 3.8 | 0.350 | 3.0 | 0.479 | 0.6 | 0.286 | 1.2 | 0.319 | 1.3 | 0.197 |
| Residual | 92 | 51.8 |  | 49.0 |  | 7.3 |  | 15.8 |  | 13.3 |  |
| Total | 119 | 100.0 |  | 100.0 |  | 100.0 |  | 100.0 |  | 100.0 |  |
|  |  | x^4^ transformed | |  | | Sqrt transformed | | Sqrt transformed  1 plant without seeds expcluded | | Sqrt transformed  1 plant without seeds expcluded | |
