# Supplementary material for: Transgene × Environment Interactions in Genetically Modified Wheat
Source: PLoS One. 2010 Jul 12;5(7):e11405. doi: 10.1371/journal.pone.0011405 (PMC2902502; doi:10.1371/journal.pone.0011405)
Supplement: Table S2 — ANOVA table of phenological state, plant height, vegetative mass, seed number and seed yield data from the glasshouse experiment. (0.05 MB DOC) [file pone.0011405.s003.doc]

**Table S2.** This ANOVA table shows the effect of the Fertilizer, GM / control, Offspring pair treatments and their interactions on the phenological state, plant height, vegetative mass, seed number, seed yield and ergot infected seeds in the field experiment.

|  |  | Phenological state | | Plant height | | Vegetative mass | | Seed number | | Seed yield | | Ergot Infection | |
| --- | --- | --- | --- | --- | --- | --- | --- | --- | --- | --- | --- | --- | --- |
| Source of variation | df | % SS | F pr. | % SS | F pr. | % SS | F pr. | % SS | F pr. | % SS | F pr. | % SS | F pr. |
| Block | 3 | 69.1 | <.001 | 3.0 | 0.583 | 3.7 | 0.511 | 3.7 | 0.073 | 4.9 | 0.035 | 2.1 | 0.229 |
| Fertilizer | 1 | 0.6 | 0.288 | 12.7 | 0.006 | 15.2 | 0.003 | 20.0 | <.001 | 19.4 | <.001 | 0.1 | 0.719 |
| GM / control | 1 | 0.0 | 0.935 | 3.7 | 0.126 | 0.1 | 0.761 | 32.3 | <.001 | 31.7 | <.001 | 40.3 | <.001 |
| Offspring pair | 3 | 3.7 | 0.083 | 1.8 | 0.759 | 3.5 | 0.538 | 7.5 | 0.004 | 3.5 | 0.097 | 20.0 | <.001 |
| GM / control x Offspring pair | 3 | 1.5 | 0.418 | 1.7 | 0.768 | 3.1 | 0.592 | 11.9 | <.001 | 14.1 | <.001 | 14.8 | <.001 |
| Fertilizer x GM / control | 1 | 0.6 | 0.288 | 0.1 | 0.754 | 0.2 | 0.702 | 0.6 | 0.271 | 0.6 | 0.284 | 0.3 | 0.415 |
| Fertilizer x Offspring pair | 3 | 0.7 | 0.720 | 6.6 | 0.239 | 1.6 | 0.806 | 0.3 | 0.908 | 0.4 | 0.852 | 0.8 | 0.625 |
| Fertilizer x GM / control x Offspring pair | 3 | 0.7 | 0.720 | 2.4 | 0.665 | 0.7 | 0.936 | 1.5 | 0.397 | 1.7 | 0.364 | 0.3 | 0.884 |
| Residual | 45 | 23.2 |  | 68.0 |  | 71.8 |  | 22.2 |  | 23.6 |  | 21.3 |  |
| Total | 63 | 100.0 |  | 100.0 |  | 100.0 |  | 100.0 |  | 100.0 |  | 100.0 |  |
|  |  | x^4^ transformed | |  | | Square root transformed | | Sqare root transformed | | Square root transformed | | Cube root transformed | |
