# Supplementary material for: Transgene × Environment Interactions in Genetically Modified Wheat
Source: PLoS One. 2010 Jul 12;5(7):e11405. doi: 10.1371/journal.pone.0011405 (PMC2902502; doi:10.1371/journal.pone.0011405)
Supplement: Table S3 — ANOVA table of phenological state, plant height, vegetative mass, seed number, seed yield and ergot infection data from the field experiment. (0.04 MB DOC) [file pone.0011405.s004.doc]

**Table S3.** These ANOVA tables shows the effect of the Fertilizer, GM / control, Offspring pair treatments and their interactions (3 way interaction omitted) on the mildew infection rate in the glasshouse and field experiment.

|  | Glasshouse | | | -------------- | Field | | |
| --- | --- | --- | --- | --- | --- | --- | --- |
| Source of variation | df | % SS | F pr. |  | df | % SS | F pr. |
| Block | 4 | 0.5 | 0.854 |  | 3 | 2.1 | 0.413 |
| Fertilizer | 2 | 3.2 | 0.016 |  | 1 | 9.8 | <.001 |
| GM / control | 1 | 48.4 | <.001 |  | 1 | 32.0 | <.001 |
| Offspring pair | 3 | 1.8 | 0.186 |  | 3 | 6.1 | 0.051 |
| GM / control x Offspring pair | 3 | 2.0 | 0.15 |  | 3 | 7.8 | 0.022 |
| Fertilizer x GM / control | 2 | 2.3 | 0.052 |  | 1 | 0.3 | 0.538 |
| Fertilizer x Offspring pair | 6 | 5.4 | 0.032 |  | 3 | 6.6 | 0.039 |
| Residual | 98 | 36.4 |  |  | 48 | 35.2 |  |
| Total | 119 | 100.0 |  |  | 63 | 100.0 |  |
|  |  |  |  |  |  |  |  |
